# Challenges in Management of Ovotesticular Differences in Sex Development in Resource-Limited Settings

**DOI:** 10.1155/2024/9987144

**Published:** 2024-04-18

**Authors:** Serwah Bonsu Asafo-Agyei, Emmanuel Ameyaw, Boateng Nimako, Michael Amoah

**Affiliations:** ^1^School of Medicine and Dentistry, Kwame Nkrumah University of Science and Technology, Private Mail Bag, University Post Office, Kumasi, Ghana; ^2^Komfo Anokye Teaching Hospital, P.O. Box 1934, Kumasi, Ghana

## Abstract

Differences in sex development (DSD) are congenital conditions in which the development of chromosomal, gonadal, or anatomical sex is atypical. Ovotesticular DSD is the rarest variant of DSD where both ovarian and testicular tissues co-exist in an individual. Ambiguous genitalia may be a glaring indicator of DSD, but multiple genital anomalies should also raise a suspicion of DSD. This is a case report of a 15-year-6-month-old boy who presented during infancy with multiple genital anomalies requiring surgery. The diagnosis of ovotesticular DSD was missed until later in adolescence when he presented with progressive bilateral breast enlargement. Work-up revealed a 46, XX karyotype and dysgenetic testes, but functional ovarian tissue. The patient wanted to consider switching to a female gender but was constrained by psychosocial factors. Maintenance of a masculine phenotype was done using testosterone injections due to the relatively high cost of testosterone patches. *Conclusion*. Multiple genital anomalies should raise the suspicion of DSD, and prompt referral to an endocrinologist should be done before urogenital surgery and gender assignment are carried out.

## 1. Introduction

Differences in sex development (DSD) are congenital conditions in which the development of chromosomal, gonadal or anatomical sex is atypical [1]. Ovotesticular DSD (OT-DSD), previously called true hermaphroditism, is a relatively rare subtype of DSD where both ovarian and testicular tissues co-exist in an individual [2]. Worldwide, it accounts for less than 10% of cases of DSD in most published literature, although it has been reported to be relatively more common in Kenya and might be the most common cause of DSD in South Africa [3–6]. Most patients present because of genital ambiguity, gynecomastia, or genital anomalies like hypospadias [7, 8]. Hospital records from our pediatric and adolescent endocrine clinic at Komfo Anokye Teaching Hospital (KATH), Ghana, indicated that while patients with genital ambiguity generally presented during infancy, those with multiple genital anomalies or gynecomastia presented later in childhood or adolescence.

Making a diagnosis of OT-DSD may be challenging in a resource-limited setting. Most physicians in resource-limited settings use the presence of palpable gonads as a surrogate marker for genetic sex since karyotyping is not readily available. It is thus assumed that if gonads are found in the labioscrotal folds or the inguinal region, then the patient most likely has a 46, XY karyotype and that guides the subsequent investigations. Invariably, this also affects sex assignment. In the authors' personal experiences, most family members and healthcare professionals tend to assume that assigned gender should be consonant with (presumed) genetic sex; placing far less emphasis on factors like definitive diagnosis, genital functionality, fertility, and psychological gender. However, this approach of using palpable gonads to suggest genetic sex may be erroneous in OT-DSD. Despite a palpable gonad, in most clinical scenarios described, the majority of patients have a 46, XX karyotype [8]. There may be asymmetry of the tissue texture at the poles of an ovotestis [8], but this may be missed during the examination. Furthermore, the diagnosis may be missed if the patient does not present with overt genital ambiguity but rather presents as an apparent male/female with isolated/multiple genital abnormalities. Isolated genital malformations are relatively common in the general population, but multiple genital abnormalities in a patient should raise clinical suspicion of DSD. DSD states have been identified in approximately 30% of patients with hypospadias and unilateral or bilateral undescended testes [9].

This case reports an adolescent brought up as a boy who had apparent male genitalia with multiple genital anomalies at birth. Three corrective surgeries were done in infancy by the adult Urologist. An endocrinology consult was not sought at that time and records of the surgeries were missing. The patient then reported during puberty on account of bilateral progressively enlarging breasts.

## 2. Case Presentation

A 15-year-6-month-old adolescent with a male gender of rearing reported to the pediatric and adolescent endocrine clinic at KATH on account of bilateral progressive breasts enlargement over nine months' duration. Symptoms of apocrine body odor with pubic hair, a growth spurt, and some enlargement of the penile and testicular size had been noted before this. KATH is the second largest tertiary hospital in Ghana, serving as the main referral center for the entire northern and middle sectors of Ghana. It has the largest pediatric and adolescent endocrine clinic in the country, manned by 2 pediatric endocrinologists.

The patient had been seen in infancy by the urologist on account of multiple genital anomalies (chordee, bilaterally undescended testes, and hypospadias). Three surgeries had been done during infancy for the repair of genital anomalies and right orchidopexy. During surgery, the left testis was not found. There was no family history of consanguinity, unexplained neonatal death, infertility, or atypical genitalia/genital malformations.

**Figure 1:**
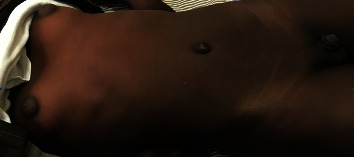
Image of patient at first clinic attendance.

Using male growth charts, anthropometry findings included a weight of 47 kg (<10^th^ centile) and a height of 154 cm (<3^rd^ centile, mid-parental height–15^th^ centile). A general examination did not reveal any dysmorphic features. Blood pressure was normal at 110/70 mmHg. The breasts were at Tanner Breast Stage 4. It was tense with a large areola. The pubic hair was estimated to be at Pubic Hair Tanner Stage 3-4 (the patient had shaved). On genital examination, only the right testis was palpable. It was fluctuant with a testicular volume of 8 mls. The labioscrotal folds looked asymmetric with the left side being poorly formed. The stretched phallic length was 5.8 cm with a single orifice at the tip. There was no vestibule.  Figure 1 shows an image of the patient at the first clinical visit.

**Figure 2:**
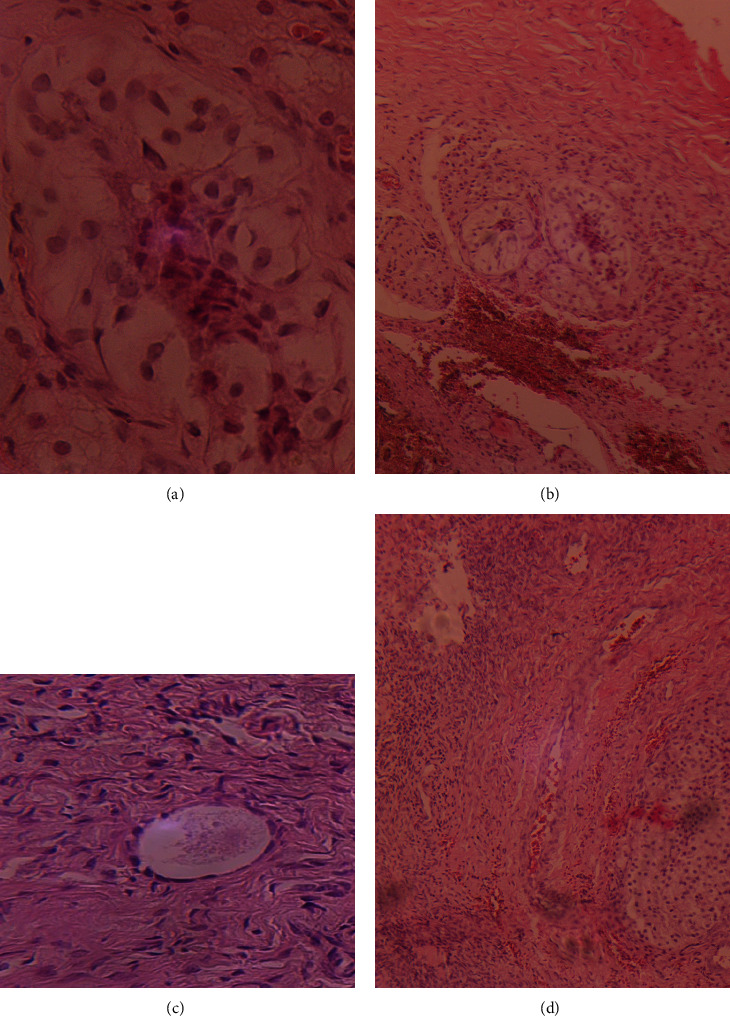
(a, b) Testicular seminiferous tubules with only sertoli cells. (c) Ovarian stroma with primordial follicle (×40 magnification). (d) Ovarian stroma and testicular tissue.

Abdominopelvic ultrasonography and magnetic resonance imaging (MRI) of the abdomen and pelvis identified a 4.9 × 1.3 × 2.7 cm cystic mass behind the bladder (Müllerian duct derivative). No gonads were seen in the abdominopelvic region. The kidneys and adrenals were normal. A labioscrotal Doppler sonography found a large right testicular cyst with absent blood flow. The left hemi-scrotum was empty and the left testis was not visualized. No prostate gland was found. Gonadotropin levels were pubertal. Estradiol levels were comparable to levels for a pubertal female. Testosterone levels were pre-ubertal with a poor response on day 4 to a single dose (5000IU) human chorionic gonadotropin (hCG) stimulation protocol. Karyotype was 46, XX in 50 metaphase cells examined. No mosaicism was found; though low-level mosaicism cannot be ruled out. Patient and parental informed consent was obtained and laparoscopic gonadal biopsies were taken from both poles of the gonad. Gonadal histopathology revealed a normal ovarian portion with degenerative changes at the testicular portion (Figures 2(a)–2(d)).  Table 1 shows further details of the investigations done.

Molecular genetics for genetic mutations, including fluorescence in situ hybridization (FISH) analysis for sex-determining region Y protein (SRY) and chromosomal analysis of gonadal tissues, was not done.

A definitive diagnosis of 46, XX ovotesticular DSD was made. The patient, together with his family, was counselled extensively by the multidisciplinary medical team including the clinical psychologist, pediatric endocrinologist, endocrine nurse educator, pediatric urologists, and plastic surgeon. Initially, the patient wanted to switch gender to that of a female on account of the ovarian tissue being functional and the financial burden of testosterone replacement therapy. However, after long deliberation, the patient and his family decided to maintain the gender of rearing on account of an already established male gender role in their community. Bilateral mammoplasty and gonadectomy with the placement of testicular prostheses have been planned. The patient will be put on testosterone injections (testosterone enanthate 200 mg per 2 weekly) afterward to maintain a male phenotypic appearance. Serum testosterone levels will be monitored (initially quarterly and later annually) and the necessary dose adjustments made.

## 3. Discussion

OT-DSD is the histological presence of both ovarian and testicular tissue in an individual, existing either separately or together within an ovotestis [2]. The most common gonad is an ovotestis, which was found in the index case [5, 8]. Karyotypes of patients with OT-DSD show marked variation with the commonest being 46, XX in most reported studies, while 46, XY, and other chimeric/mosaic forms are relatively uncommon [5, 8]. In some patients with a 46, XX karyotype, the testicular tissue results from translocation of the SRY gene to the X chromosome or another chromosome [2]. The index patient had a 46, XX karyotype, but we were unable to carry out an SRY FISH analysis due to financial constraints.

Genital appearance in OT-DSD varies and may range from overt genital ambiguity to what may be interpreted as genital malformations in an apparent male/female. Clinical clues to the diagnosis are particularly important in resource-limited settings because of the lack of neonatal screening programs and the unavailability/prohibitive cost of laboratory investigations. Genital ambiguity is a glaring indicator of the presence of a DSD. A subtle pointer may be the presence of multiple genital anomalies. Isolated genital anomalies are relatively common in the general population but multiple genital anomalies should increase the suspicion of a DSD [9]. The index patient presented to an adult urologist with multiple genital anomalies (bilateral cryptorchidism, chordee, and hypospadias) which should have prompted further investigations or a pediatric endocrinologist consult. Different tissue textures of a gonad may be suggestive of an ovotestis, which may have a soft testicular portion and a firm ovarian portion [8].

The development of breasts in a patient reared as male has also been found to be an important presenting symptom [10]. In most patients with OT-DSD, the testicular tissue progressively regresses and becomes dysgenetic with interstitial fibrosis and loss of spermatogonia, while the ovarian tissue usually remains functional [11]. Fertility as a female is thus possible and pregnancies have been reported, whereas fertility as a male is highly unlikely [12]. Pubertal onset may be associated with breast enlargement due to estrogen production from the functional ovarian tissue, in contrast to diminishing testosterone synthesis from progressive testicular regression. In our patient, it was the progressive breast enlargement that prompted the patient to seek medical attention.

The diagnosis and subsequent management of DSDs requires a targeted and structured approach, involving an interactive multidisciplinary team with adequate psychological support. This includes clinical, imaging, laboratory, and genetic evaluations [13]. Evaluations should be done efficiently to expedite gender assignment while avoiding unnecessary costs. Close monitoring for potentially life-threatening complications like malignancies should be promptly recognized and treated and necessary medical, surgical, and psychological interventions should be carried out seamlessly [14].

It is always important to determine the underlying cause of DSD as this may impact management and sex assignment. A pragmatic approach in resource-limited settings is to conduct a thorough clinical assessment; paying attention to the presence, position, and texture of the gonads. Requesting for a battery of investigations simultaneously may be financially challenging for patients as well as unproductive. Abdominopelvic and labioscrotal ultrasonography by skilled personnel is a relatively inexpensive and noninvasive first step in seeking direction about how to proceed with further investigations. Other imaging modalities like MRI and genitogram may better delineate the internal anatomy and gonads [15]. The combination of both male and female internal and external phenotypes is suggestive of OT-DSD [16].

Karyotyping, if available and affordable, can be invaluable in narrowing down possible differential diagnoses when combined with detailed clinical assessment and imaging findings. The majority of virilized 46, XX children will likely have congenital adrenal hyperplasia. The presence of labioscrotal/inguinal gonads, uterine anomalies/dysplasia, or a normal adrenal steroid profile should raise suspicion of OT-DSD [13]. Despite this, a definitive diagnosis may be reached without doing karyotyping. Other genetic tests including FISH analysis or polymerase chain reaction for Y chromosome or genetic sequencing are costly and largely unavailable in most resource-limited settings. Basal/stimulated serum levels of testosterone, estradiol, gonadotrophins, and basal serum anti-müllerian hormone (AMH) may be used to assess for functional gonadal tissue [16, 17]. Gonadotropins and estradiol levels in OT-DSD are usually appropriate for the pubertal level, whereas testosterone and AMH levels may vary depending on the degree of testicular regression [16]. Gonadal histopathologic analysis confirms OT-DSD and excludes other differential diagnoses, especially mixed gonadal dysgenesis which has a similar clinical and cytogenetic presentation [18].

Gender assignments can be fraught with challenges. If the diagnosis is established early, gender assignment may be influenced by fertility and functional potential as well as phenotypic sex. Gender reassignment is usually not done if the diagnosis is made late, but management should still be individualized to suit the patient [19]. The index patient and his family deliberated for a long time before making an informed decision to maintain the gender of rearing. The option of maintaining a male phenotype with testosterone injections, relative to switching to a female gender, was more expensive and inconvenient. In our setting, testosterone patches/gels are largely unavailable and expensive. Alternatively, they were also worried about the potential stigma if the child had to switch gender at this age. This further emphasizes the necessity of making the diagnosis early to give the family more freedom to choose the gender of rearing based on the prevailing circumstances.

Surgical management consists of surgical correction of medically concerning issues like malignancy, shaping of the internal and external genitalia to the desired gender with preservation of gonadal tissue concordant with the sex of rearing, and removal of all discordant tissue [16].

Unlike patients with mixed gonadal dysgenesis and pure gonadal dysgenesis, the risk of malignancy in OT-DSD is much lower. The slightly increased risk of malignancy in OT-DSD likely stems from the ectopic position of dysgenetic testicular tissue, as well as an increased risk of gonadoblastoma in dysgenetic gonads with Yp material [16, 20]. Other reported complications in males include urethral fistula and late urethral stenosis [21].

## 4. Conclusion

This case report will help improve the awareness of clinicians and the general public about the myriad forms of presentation of OT-DSD. DSDs, including OT-DSD, should be suspected when there are multiple genital malformations. Apart from ambiguous genitalia/multiple genital anomalies, OT-DSD can also present in adolescence as progressive bilateral gynecomastia. Improved awareness will result in earlier diagnosis and subsequently aid in the prevention of gender dysphoria and stigmatization, with improved parental acceptance of their children with DSD.

The management of OT-DSD can be especially challenging in resource-limited settings. Special considerations include the financial burden of investigations and treatment, the unavailability of some diagnostic modalities, and entrenched socio-cultural norms stigmatizing sexuality issues. The management of a patient with OT-DSD must therefore be carried out seamlessly by a multidisciplinary team in a socio-culturally sensitive manner to achieve the most yields with minimal cost. A genetic counselor as a member of the multi-disciplinary team is needed.

## Figures and Tables

**Table 1:** Results of investigations.

Laboratory investigation	Results	^*∗*^Normal range
Luteinizing hormone (U/L)	6.04	2.0–8.0
Follicular stimulating hormone (IU/L)	4.17	3.0–5.5
Estradiol (pmol/l)	363.6	<150
Testosterone (nmol/l)	3.9	10.0–30.0
hCG stimulation test (single dose protocol)-increment in testosterone (%)	37	200–300
Gonadal biopsy histopathology	Both ovarian follicles and testicular tubular elements seen with connective tissue separating them. Ovarian portion appeared normal with fallopian tube attached. Testicular portion showed degenerative and cystic changes; spermatic cord attached. No evidence of malignancy	

^*∗*^Normal range denotes the value for a pubertal 15-year-6-months-old male.

## Data Availability

No data were used to support this research.

## References

[1] Hughes I., Houk C., Ahmed S., Lee P. (2006). Consensus statement on management of intersex disorders. *Journal of Pediatric Urology*.

[2] Cools M., Kohler B., Dattani M., Brook C. (2020). Disorders of sex development. *Brook’s Clinical Pediatric Endocrinology*.

[3] Jaruratanasirikul S., Engchaun V. (2014). Management of children with disorders of sex development: 20-year experience in southern Thailand. *World J Pediatr*.

[4] Amolo P., Laigong P., Omar A., Drop S. (2019). Etiology and clinical presentation of disorders of sex development in Kenyan children and adolescents. *International Journal of Endocrinology*.

[5] Ganie Y., Aldous C., Balakrishna Y., Wiersma R. (2017). The spectrum of ovotesticular disorders of sex development in South Africa: a single-centre experience. *Hormone Research in Paediatrícs*.

[6] Ganie Y., Aldous C., Balakrishna Y., Wiersma R. (2017). Disorders of sex development in children in KwaZulu-Natal Durban South Africa: 20-year experience in a tertiary centre. *Journal of Pediatric Endocrinology & Metabolism: Journal of Pediatric Endocrinology & Metabolism*.

[7] Kim Y. M., Oh A., Kim K. S., Yoo H. W., Choi J. H. (2019). Pubertal outcomes and sex of rearing of patients with ovotesticular disorder of sex development and mixed gonadal dysgenesis. *Annals of Pediatric Endocrinology and Metabolism*.

[8] Mao Y., Chen S., Wang R., Wang X., Qin D., Tang Y. (2017). Evaluation and treatment for ovotesticular disorder of sex development (OT-DSD)-experience based on a Chinese series. *BMC Urology*.

[9] Kaefer M., Diamond D., Hendren W. H. (1999). The incidence of intersexuality in children with cryptorchidism and hypospadias: stratification based on gonadal palpability and meatal position. *The Journal of Urology*.

[10] Özdemir M., Kavak R. P., Yalcinkaya I., Guresci K. (2019). Ovotesticular disorder of sex development: an unusual presentation. *Journal of Clinical Imaging Science*.

[11] Verkauskas G., Jaubert F., Lortat-Jacob S., Malan V., Thibaud E., Nihoul-Fékété C. (2007). The long-term followup of 33 cases of true hermaphroditism: a 40-year experience with conservative gonadal surgery. *The Journal of Urology*.

[12] Krob G., Braun A., Kuhnle U. (1994). True hermaphroditism: geographical distribution, clinical findings, chromosomes and gonadal histology. *European Journal of Pediatrics*.

[13] Hiort O., Birnbaum W., Marshall L. (2014). Management of disorders of sex development. *Nature Reviews Endocrinology*.

[14] Swain S., Pradhan L., Satpathy R. N., Mahapatra P. C. (2014). A rare case report on ovotesticular disorders of sex development (DSD) 46XY variant. *International Journal of J Reprod Contracept Obstet Gynecol*.

[15] Al Jurayyan N. A., Al-Jurayyan R. N. A., Mohamed S. H., Babiker A. M., Al Otaibi H. M. (2013). Radiological imaging of disorders of sex development (DSD). *Sudan J Paediatr*.

[16] Vilain E., Sarafoglou K., Yehya N., Sarafoglou K., Hoffmann G., Roth K. S., Courtney H. (2009). Disorders of sex development. *Pediatric Endocrinology and Inborn Errors of Metabolism*.

[17] Josso N., Rey R. A. (2020). What does AMH tell us in pediatric disorders of sex development?. *Frontiers in Endocrinology*.

[18] Fine S., Ford K., Trotter B. (2021). Mixed gonadal dysgenesis with an ovotestis on imaging mimicking ovotesticular disorder of sexual differentiation. *Baylor University Medical Center Proceedings*.

[19] Fisher A. D., Ristori J., Fanni E., Castellini G., Forti G., Maggi M. (2016). Gender identity, gender assignment and reassignment in individuals with disorders of sex development: a major of dilemma. *Journal of Endocrinological Investigation*.

[20] Cools M., Drop S. L. S., Wolffenbuttel K. P., Oosterhuis J. W., Looijenga L. H. J. (2006). Germ cell tumors in the intersex gonad: old paths, new directions, moving frontiers. *Endocrine Reviews*.

[21] Sircili M. H. P., Denes F. T., Costa E. M. F. (2014). Long-Term followup of a large cohort of patients with ovotesticular disorder of sex development. *The Journal of Urology*.

